# Prevalence of intestinal parasites among HIV patients at the Yaoundé Central Hospital, Cameroon

**DOI:** 10.11604/pamj.2014.18.136.3052

**Published:** 2014-06-11

**Authors:** Marius Zambou Vouking, Patrice Enoka, Claire Violette Tamo, Carine Nouboudem Tadenfok

**Affiliations:** 1 Center for the Development of Best Practices in Health, Yaoundé Central Hospital, Yaoundé, Cameroon; 2 Centre Regional Delegation, Ministry of Public Health, Yaoundé, Cameroon; 3 ‘Ecole des Techniciens Médico-Sanitaires de Yaoundé’, Cameroon; 4 Catholic University for Central Africa School of Health Sciences, Yaoundé, Cameroon

**Keywords:** Prevalence, intestinal parasites, HIV, Yaoundé Central Hospital Accredited Treatment Centre, Cameroon

## Abstract

**Introduction:**

Intestinal parasites are more common in people with HIV, especially in tropical developing countries. This cross-sectional study was carried out to assess the prevalence of intestinal parasites among people with HIV at the Yaoundé Central Hospital Accredited Treatment Centre.

**Methods:**

Structured questionnaires were used to collect clinical information after obtaining consent from the participants. Stool samples were collected from 207 HIV-positive patients for the investigation of intestinal pathogens using direct microscopy, formalin-ether concentration, ZiehlNeelsen modified and Kato-Katz methods. Data was analyzed using Epi-info version 3.4.1. and Microsoft Office Excel 2007.

**Results:**

A total of 207 people were recruited. Eighty (38.65%) were male and 127 (61.35%) were female. The overall prevalence of intestinal parasite infections was 57.48% (119/207). The parasites detected in our study population included *Entamoeba coli* (22.68%), *Ascaris lumbricoïdes* (22.68%), *Entamoeba histolytica*(15.93%), *Cryptosporidium spp* (12.60%), *Isospora belli* (10.08%), *Trichuris trichiura* (7.60%), *Strongyloïdesstercoralis* (5.88%), *Ancylostomaduodenale* and *Necatoramericanus* (2.52%).

**Conclusion:**

At the end of our study, it appears that intestinal parasites still occupy an important place among HIV-positive patients.

## Introduction

Human Immunodeficiency Virus/Acquired Immune Deficiency Syndrome (HIV/AIDS) is a global pandemic and has been reported in almost every country of the world [1]. About 66% (22 million) of the estimated 34 million infected people are in sub-Saharan Africa are living with HIV/AIDS [1].

Intestinal parasitic infections which are caused either by protozoa or helminths or both are among the most widespread of human infections worldwide. It is estimated that as much as 60% of the World's population is infected with intestinal parasites which may play a significant role in morbidity due to intestinal infections [2]. The rate of infection is also remarkably high in sub-Saharan Africa, where the majority of people with HIV/AIDS are concentrated where factors including poverty and malnutrition could promote transmission of both infections in the region [3].

Immunosuppression, a consequence of HIV infection favors the occurrence of multiple opportunistic infections responsible for a high mortality [4, 5]. Among these diseases, intestinal parasites are the main cause of severe chronic diarrhea [6]. *Coccidia*, *Cryptosporidium parvum, Isospora belli, Cyclosporaspp* and *Entamoeba histolytica* are the etiologic agents commonly responsible for the genesis of these intestinal protozoans in HIV-positive persons in many parts of the world [7].

Most helminth infections also induce strong immunoregulatory responses driven by regulatory T cells, which are potentially capable of contributing to HIV pathogenesis by suppressing HIV-specific immune responses, as recently shown in vitro [8]. Few studies have addressed the issue of intestinal parasites among HIV-infected persons in Cameroon [9–11]. Our study aimed at assessing the prevalence of intestinal parasites in HIV-infected patients, at the Yaoundé Central Hospital (YCH) Accredited Treatment Centre (ATC).

## Methods

### Ethical approvals

The study was approved by the Institutional Ethical Review Board of the Yaoundé Central hospital. Permission was obtained from the administrators of the Yaoundé Central hospital. We obtained verbal consent from all patients who volunteered to participate in the study.

### Study area and population

The study was conducted at the Yaoundé Central Hospital (YCH) Accredited Treatment Centre (ATC). This is an urban centre in the heartof the capital city, Yaoundé. The YCH is a tertiary levelgeneral teaching hospital with a capacity of 381 beds. It employs nearly 800 staff including 95 doctors and 270 nurses [12]. The ACT has a very high recruitment rate of approximately 40 new cases per week and caters for 6500 regular clients.

### Process of the survey

Patients were enrolled from January to June 2007. Patients coming to the hospitals were requested to participate in the study. The inclusion criteria were: HIV-positive patients, with or without signs of diarrhea and willing to participate in the study. Following their consent, a questionnaire was administered to each participant by the nurse who had been specifically trained for this task. It contains three sections which include: Demographic and socio-economic information (age, gender, residence, location and family size), health (history of intestinal parasitic infection and diarrhea), and environmental factors (housing conditions and water supply). The exclusion criteria include: those on specific antihelminthics or who had any treatment for intestinal parasitism in the last two weeks preceding specimen collection and those who had antacid, in the last two weeks preceding specimen collection were all excluded from the study because the drugs could have killed or inhibit reproduction of the parasites.

A sample pair of stool was collected from each of the participants and used for intestinal parasites.

### Laboratory analysis

Stool specimen was examined macroscopically for consistency, mucus and blood. Stool samples were analyzed in three ways. First, a direct wet mount in physiological saline and iodine, was examined under a light microscope for ova, cyst and parasite detection. Second, samples were concentrated using a formol-ether and Kato-Katz technique [13]. Fecal smears were then stained following the ZiehlNeelsen modified technique for the detection oocysts [14]. Two trained senior medical laboratory technologists examined the samples microscopically.

### Statistical analysis

The data obtained from questionnaires was analyzed using the statistical software Epi-info version 3.4.1. and Excel 2007. The information obtained from the questionnaire was presented as percentages and means. The results obtained from the stool specimens were presented as frequency and percentage.

## Results

### Baseline characteristics of participants

A total of 207 people were recruited, 80 (38.65%) were male and 127 (61.35%) were female. Sex ratio M/F was 0.62. One hundred and ninety-one (92.28%) participants were aged between 26 to 50 years. Eighty (38.65) of the 207 stool samples presented a diarrhoeic aspect. The summary of these data are presented in Table 1.

**Table 1:** Baseline characteristics of participants

Characteristic		Number (%)
**Age**	≤ 25	9 (4.34)
	[26 - 50]	191 (92.28)
	> 50	7 (3.38)
**Mean age ± SD (years)**	36,06 ±09 years	
**Sex**	Male	80 (38.65)
	Female	127 (61.35)
**Antiretroviral treatment**	Yes	107 (51.70)
	No	100 (48.30)
**Hospitalized**	Yes	80 (38.65)
	No	127 (61.35)
**Place of residence**	Urban	137 (66.18)
	Rural	70 (33.82)
**Diarrhea**	Yes	80 (38.65)
	No	127 (61.35)

### The prevalence of infection with respondent's characteristics

The overall prevalence of intestinal parasite infections was 57.48% (119/207) (Table 2). The parasites detected in our study population included *Entamoeba coli* (22.68%), *Ascaris lumbricoïdes* (22.68%), *Entamoebahistolytica* (15.93%), *Cryptosporidium spp* (12.60%), *Isospora belli* (10.08%), *Trichuris trichiura* (7.60%), *Strongyloïdesstercoralis* (5.88%), *Ancylostomaduodenale* and *Necatoramericanus* (2.52%) (Table 2).

**Table 2:** Distribution of intestinal parasites among HIV positive patients

Parasite	Frequency of parasites	Proportion (%)
*E. coli*	27	22.68
*A. lumbricoïdes*	27	22.68
*E. histolytica*	19	15.93
*Cryptosporidium spp*	15	12.60
*I. belli*	12	10.08
*T. trichiura*	9	7.60
*S. stercoralis*	7	5.88
*A. duodenale, N. americanus*	3	2.52
**Total**	119	100

## Discussion

Our study aimed at finding the prevalence of HIV-intestinal parasites co-infection at the YCH-ATC and to evaluate the risk factors associated to this co-infection. The overall prevalence of intestinal parasites was high as 57.48% in the HIV infected patients. Our data showed that the major risk factors for intestinal parasitosis were the HIV status and the quality of water consumed. This result confirms the observations of many authors that intestinal parasites occupy an important place among the gastrointestinal tract diseases in people generally and those infected with HIV in particular in developing countries [15–18].

The prevalence of intestinal parasites in Cameroon varies from 33% in 2006 [9] to 27.8% in 2012 [10] and to 57.48% in the present study. This higher rate may be explained by the use of two methods of concentration (that of Kato and the Ritchie) specific both to helminths and protozoan research, which was not the case in three other studies that used a single concentration method (Ritchie) specific to protozoan.

Intestinal parasites are a diverse group of microorganisms that include single-celled protozoans and multi-cellular intestinal worms, capable of disrupting the absorption of nutrients.

In Addition, apart from the effects of HIV enteropathy, the presence of intestinal parasites is an important contributing factor to diarrhoea, which in the longer term could result in the wasting syndrome called “slim disease” [19].

The most found Protozoan were *E. coli* and *E. histolytica* with respective rates of 22.68% and 15.95%. These results also confirm those of Zeynudin et al. [20] in Ethiopia, Gassama et al. [19] in Senegal who found almost similar rates of 15.10% and 19.60% respectively for *E. histolytica* and *E. coli* and concluded that amibiasis was the main non-opportunistic protozoa in HIV-positive subjects.

Among the known opportunistic intestinal parasites, *Crystosporidiumspp* was encountered with the frequency of 12.60%. These results are higher than those of Lehman et al. 2012 [10] obtained in Douala- Cameroon where *C. spp* was 7.4%, and those reported by Safarti et al. 2006 in Yaounde-Cameroon (3.9%) [9]. *Isospora belli* was reported with the frequency of 10.08%. This high prevalence is in line with the study done in Thailand which reported 5% prevalence (Silva et al., 2005), 7% in Brazil [16] and 8% in Venezuela [9]. The most frequently encountered helminths eggs are those of *A. lumbricoides* and *T. trichiura* followed by the larvae of *S. stercoralis* at respective rates of 22.68%, 7.60%, 5.88%. These results are in agreement with those obtained by Modjarrad et al. [21] in Lusaka who found respective rates of 25.63%, 5.30%, 5.40% in HIV positive subjects. These authors concluded that, apart from *S. stercoralis*, other helminths do not have a particular increase in HIV-positive subjects, but they can seriously disrupt their feeding and influence their care especially those on ARTs. A systematic review has shown that approximately one-third to one-half of the global population is infected with at least one species of helminth, with the vast majority of these infections occurring in resource-limited areas of the world where the HIV/AIDS pandemic is most severe [19]. This review suggests that treatment of helminth infection may result in delayed progression of HIV-1 disease as measured by change in CD4 counts and plasma viral load [9]. Our study was also able to reaffirm the importance of concentration methods in the stool diagnosis of intestinal parasites. In underdeveloped countries the opportunity to integrate the systematic coprology coupled with the use of concentration techniques in the comprehensive care of people living with HIV is under consideration [9–11, 21].

## Conclusion

At the end of our study, it appears that intestinal parasites still occupy an important place among HIV-positive patients. Dirty water promotes these intestinal parasites. The recurrent nature of these infections highlights a relationship with the condition insofar as intestinal parasites could alter their condition and complicate their treatment by the disruption of their diet.

## References

[1] WHO (2011). Global HIV/AIDS response: epidemic update and health sector progress towards universal access: progress report 201. http://www.who.int.

[2] (1987). World Health Organization. Prevention and control of intestinal parasitic infections. WHO Technical Report.

[3] UNAIDS/WHO (2002). HIV Epidemic Update.

[4] Konaté A, Minta D, Diarra M, Dolo A, Dembele M (2005). Parasitoses digestives au cours de la diarrhée du sida. Bull Soc PatholExot..

[5] Wumba R, Enache-Angoulvant A, Develoux M, Mulumba A, Mulumba PM (2007). Prévalence des infections opportunistes digestives parasitaires à Kinshasa (République Démogratique du Congo), Résultats d'une enquête préliminaire chez 50 patients au stade SIDA. Med Trop..

[6] Assefa S, Erko B, Medhin G, Assefa Z, Shimelis T (2009). Intestinal parasitic infections in relation to HIV/AIDS status, diarrhea and CD4 T-cell count. BMC Infect Dis..

[7] Ekejindu IM, Ele PU, Okonkwo SO, Ezenwagu OC, Ezeagwuna DA (2010). Intestinal parasitic infection among HIV-seropositive and HIV-seronegative individuals at Nnewi, South Eastern Nigeria. World Journal of Medical Sciences..

[8] Kinter AL, Horak R, Sion M (2007). CD25+ regulatory T cells isolated from HIV-infected individuals suppress the cytolytic and nonlytic antiviral activity of HIV-specific CD8+ T cells in vitro. AIDS Res Hum Retroviruses..

[9] Sarfati C, Bourgeois A, Menotti J, Liegeois F, Moyou-Somo R (2006). Prevalence of intestinal parasites including microsporidia in human immunodeficiency virus-infected adults in Cameroon: A cross-sectional study. American Journal of Tropical Medicine and Hygiene..

[10] Lehman LG, Kangam L, Nguepi E, Mbenoun ML, BilongBilong CF (2012). Study of intestinal parasitic infections associated with HIV infection in Douala, Cameroon. Retrovirology..

[11] Nkenfou CN, Nana CT, Payne VK (2013). Intestinal Parasitic Infections in HIV Infected and Non-Infected Patients in a Low HIV Prevalence Region, West-Cameroon. PLoS ONE.

[12] (2010). http://www.who.int/patientsafety/implementation/apps/first_wave/cameroon_hug/en/.

[13] Levine JA, Estevez GE (1983). Method for Concentration of Parasites from Small Amounts of Feces. J ClinMicrobiol..

[14] Tuli L, Singh Deepak K, Gulati Anil K, Shyam Sundar, Tribhuban M Mohapatra (2010). A multiattribute utility evaluation of different methods for the detection of enteric protozoa causing diarrhea in AIDS patients. BMC Microbiol.

[15] Alfonso Y, Monzote L (2011). HIV Protease Inhibitors: Effect on the Opportunistic Protozoan Parasites. Open Med Chem..

[16] Tian LG, Chen JX, Wang TP, Cheng GJ, Steinmann P (2012). Co-infection of HIV and intestinal parasites in rural area of China. Parasites &Vectors..

[17] Walson JL, Herrin BR, John-Stewart G (2009). Deworming helminth co-infected individuals for delaying HIV disease progression. Cochrane Database of Systematic Reviews.

[18] Chacin-Bonilla L, Panunzio AP, Monsalve-Castillo FM, Parra-Cepeda IE, Martinez R (2006). Microsporidiosis in Venezuela: prevalence of intestinal microsporidiosis and its contribution to diarrhea in a group of human immunodeficiency virus-infected patients from Zulia State. Am J Trop Med Hyg..

[19] Gassama A, Thiaw B, Dia NM, Fall F, Camara P, Hovette P, Perret JL, Gueye-Ndiaye A, Mboup S, Sow PS, Aidara-Kane A (2001). Infective etiology of diarrhea in adults with HIV infection in Dakar: a case-control study on 594 patients. Dakar Med..

[20] Zeynudin A, Hemalatha K, Kannan S (2013). Prevalence of opportunistic intestinal parasitic infection among HIV infected patients who are taking antiretroviral treatment at Jimma Health Center, Jimma, Ethiopia. EurRev Med PharmacolSci..

[21] Modjarrad K, Zulu I, Redden DT, Njobvu L, Freedman DO, Vermund SH (2005). Prevalence and Predictors of Intestinal Helminth Infections Among Human Immunodeficiency Virus Type 1-Infected Adults in an Urban African Setting. Am J Trop Med Hyg..

